# Information Wanted about Coca

**Published:** 1898-03

**Authors:** W. Golden Mortimer

**Affiliations:** 504 W. 146th Street; New York


### INFORMATION WANTED ABOUT COCA.

NEW YORK, January 17, 1898.

*Atlanta Medical and Surgical Journal:*

For some time I have been preparing a work on Coca which will aim to exhaustively present, in an impartial manner, all that is known of this remarkable plant and its application.

The marvelous tales, with which we are familiar, of the wonderful sustaining powers of Coca, would alone indicate some inherent inestimable value. But either from prejudice or neglect, possibly from the greater interest directed to its alkaloid, Coca has been overlooked and has not received that attention to which through its physiological importance it is entitled.

To supplement data already at hand for this work, I addressed many representative physicians, asking their experience with Coca. While this collective investigation was commenced on the supposition that the remedy was little known and less understood, the replies have impressively emphasized this. They also have generally expressed a desire to more fully learn the true properties of Coca.

I ask that you will kindly further this work by answering the inclosed queries, and also by calling the attention of your readers to this inquiry of common interest, with a request to forward to me the result of personal observation or experience in the use of Coca, as a therapeutic agent or as a food. Very sincerely,

504 W. 146th Street. W. GOLDEN MORTIMER, M.D.

[Dr. Mortimer will be pleased to forward question-blanks to any one who will aid him in this investigation.—EDS.]

---

THE late Madame Boucicault, of Paris, has left a large portion of her estate for the erection and maintenance of a hospital for the treatment of persons affected with tuberculosis. The buildings, with furniture, have cost about $650,000, and a sum of $1,500,000 was left for its maintenance. The new hospital is isolated and situated in that part of the city known as Javel, which is on the south side of the Seine. The wards are glass-roofed galleries, the sides of which may be entirely open. This is for the air treatment, which is a method constantly growing in favor. The flooring is of polished hard wood, the furniture of enameled iron, and there is no drapery or curtain of any kind. The object is to be able to disinfect the entire apartment by means of steam and hot water. The matter of sterilization of all matter from the patients' mouths and throats is carefully looked after. Incurable patients are to be isolated in smaller rooms away from the wards.—*Medical Age.*

